# Temperatures in Excess of Critical Thresholds Threaten Nestling Growth and Survival in A Rapidly-Warming Arid Savanna: A Study of Common Fiscals

**DOI:** 10.1371/journal.pone.0074613

**Published:** 2013-09-09

**Authors:** Susan J. Cunningham, Rowan O. Martin, Carryn L. Hojem, Philip A. R. Hockey

**Affiliations:** Percy FitzPatrick Institute, DST/NRF Centre of Excellence, University of Cape Town, Rondebosch, South Africa; Southern Illinois University, United States of America

## Abstract

Frequency, duration, and intensity of hot-weather events are all predicted to increase with climate warming. Despite this, mechanisms by which temperature increases affect individual fitness and drive population-level changes are poorly understood. We investigated the link between daily maximum air temperature (t_max_) and breeding success of Kalahari common fiscals (*Lanius collaris*) in terms of the daily effect on nestling body-mass gain, and the cumulative effect on size and age of fledglings. High t_max_ reduced mass gain of younger, but not older nestlings and *average* nestling-period t_max_ did not affect fledgling size. Instead, the frequency with which t_max_ exceeded critical thresholds (t_crit_s) significantly reduced fledging body mass (t_crit_ = 33°C) and tarsus length (t_crit_ = 37°C), as well as delaying fledging (t_crit_ = 35°C). Nest failure risk was 4.2% per day therefore delays reduced fledging probability. Smaller size at fledging often correlates with reduced lifetime fitness and might also underlie documented adult body-size reductions in desert birds in relation to climate warming. Temperature thresholds above which organisms incur fitness costs are probably common, as physiological responses to temperature are non-linear. Understanding the shape of the relationship between temperature and fitness has implications for our ability to predict species’ responses to climate change.

## Introduction

Climate change is causing range shifts and local extinctions of species worldwide [1]–[3], particularly at the ‘warm edges’ of their ranges [4]. Accurately predicting and understanding such changes relies upon knowledge of the mechanistic links between climate and key biological processes [5]–[7]. Empirical data on how organisms respond to climatic variation and the implications for fitness are therefore of great importance [8].

Animals living in hot, arid environments routinely face harsh climatic conditions, including environmental temperatures with the potential to induce lethal hyperthermia (see [9]–[11] for examples of mass mortalities of bats and birds during heat-waves). Endotherms respond to such conditions by making physiological adjustments to facilitate greater heat dissipation. These include increasing evaporative water loss or undertaking facultative hyperthermia [12]. Alternatively or concurrently, they may make behavioural adjustments to lower heat load, including reducing activity and shifting into the shade [13]. Such adjustments should be non-linear in nature, as energy and water requirements for thermoregulation vary little when ambient temperatures fall within an endotherm’s thermoneutral zone (TNZ), but increase dramatically outside of it [12], [14], [15]. Furthermore, evaporative cooling becomes the only means of heat-dissipation when air temperature exceeds body temperature [15]. This results in sharp increases in water loss with associated danger of dehydration, especially for small animals [16], [17]. Thermoregulation at temperatures above the TNZ therefore carries high water and energy costs, potentially accompanied by reduced intake [7], [18] and missed-opportunity costs inherent in behavioural adjustments.

High temperatures can influence survival directly, or can have more subtle effects on fitness. For example, under high temperature conditions in the Kalahari, southern pied babblers (*Turdoides bicolor*) traded-off heat dissipation behaviours against foraging efficiency. This led to reduced mass gain, with potential implications for fitness [18]. Exposure to high temperatures also reduced long-term survival in desert-dwelling banner-tailed kangaroo rats [19]. Arid-zones globally are predicted to experience increasing frequencies and duration of hot-weather events under climate change [20], [21], potentially exposing animal populations to chronic and perhaps cumulative fitness costs [7]. Indeed, rising temperatures in Mexico have already driven declines and local extinctions of *Scleroporus* lizard populations via sub-lethal effects on activity patterns [3].

Sub-lethal costs of high temperatures may be particularly acute during breeding in species with appreciable levels of parental care. This is because adults carry the cost of providing for dependent offspring as well as for themselves, resulting in conflicts during times of resource bottlenecks (e.g. when temperature reduces the ability of parents to forage via imposing thermoregulatory costs or reducing prey availability). Reduced provisioning rates to nests during periods of high temperatures have been documented in a number of arid-zone passerine species [22]–[24]. High temperatures may also reduce growth rates in young birds under conditions of *ad libitum* food availability [25], possibly due to increased thermoregulatory demands. Nestlings therefore face a double challenge during hot weather: coping with reduced parental care at a time when their own thermoregulatory costs are high.

We studied effects of high daily maximum air temperature (t_max_) on aspects of breeding success in a species near the ‘warm edge’ of its range: the common fiscal (*Lanius collaris*) in the southern Kalahari. The common fiscal is widespread in sub-Saharan Africa including the periphery of the Kalahari, but largely absent in the central Kalahari basin [26]. It is a typical Laniidae shrike, hunting invertebrates and small vertebrates from exposed perches [27], leaving it vulnerable to high levels of solar radiation. We hypothesised that higher t_max_ would therefore negatively affect nest provisioning rates. Reduced provisioning may in turn result in lower rates of body mass gain by nestlings, with implications for size and age at fledging. As air temperatures are likely to correlate with nest temperatures (shown to affect nestling growth in other birds e.g. [28]), we further hypothesised that t_max_ would affect nestling body mass directly. We examined these effects on broods at different stages, because the effect of temperature can be modified by nestling age [28], [29].Due to the non-linear nature of physiological responses to temperature, we explored the existence of t_max_ thresholds above which the size of effects on fledging parameters began to increase. Finally, we calculated the implications of delayed fledging for nestling survival in terms of increased vulnerability to time-dependant mortality [30]..

Our aim was to provide data on links between climate, specifically temperature, and breeding success for a common bird with a wide geographical range. This data can inform predictions of how absolute fitness and population persistence may change under scenarios of global warming [7]. We used t_max_ as our standard measure of temperature because (a) we were interested in the effects of temperature at the scale of days, (b) t_max_ is likely to correlate with the range of environmental temperatures (*sensu* [31]) available to birds and can be used as an index of such (e.g. see [3], [18]), and (c) t_max_ is collected at weather stations globally and is commonly used in climate change analyses (e.g. [32]).

## Materials and Methods

### Ethics statement

The methods used in this study were approved by the University of Cape Town Animal Ethics Committee (clearance # 2011/V21/PH). The study was carried out on private land (Tswalu Kalahari Reserve) with permission of the landowners and of the Northern Cape Conservancy of South Africa (permit # Fauna 1088/2011). Bird banding was done by individuals licensed by the South African Bird Ringing Unit (SAFRING) and all bird handling was done by experienced individuals.

### Study site

We worked in a 10 km^2^ area of dunefield on Tswalu Kalahari Reserve (100 000 ha; 27°13'S, 22°22'E), South Africa. Vegetation was typical of Kalahari arid savanna, consisting of sparse grasses (*Eragrostis spp., Panicum spp., Aristida spp.*) dotted with trees and shrubs (*Acacia erioloba*, *A.haematoxylin*, *A. mellifera*, *Boscia albitrunca*, *Terminalia sericea*, *Rhigozum trichotomum*).

The study was done over two austral summers: November 2010 – March 2011 and November 2011 – February 2012, which corresponds with the breeding season for common fiscals. Air temperature maximum and minimum were 38.7°C and 8.7°C in 2010/11 and 39.1°C and 9.3°C in 2011/12, respectively. Average daily rainfall was 4 mm in 2010/11 and 3 mm in 2011/12. Rain fall was erratic with most falling heavily during thunderstorms interspersed with long dry periods. Meteorological data were collected using an onsite weather station (VantagePro 2, Davis Instruments, Hayward, California).

### Study species and population

The common fiscal is a medium-sized passerine (35–45 g) endemic to Africa. The Kalahari population is often considered a subspecies (*L. c. subcoronatus*) but recent genetic analysis does not support this distinction [33]. Common fiscals are territorial, socially monogamous, open cup breeders [27]. Pairs defended territories of ∼3 – 10 ha at our study site.

We captured fifty-four individuals (28 males, 26 females) using springtraps baited with giant mealworms (*Zophobas morio*). We sexed the birds using presence/absence of a rufous flank patch (present only in females), and fitted them with three plastic colour bands (JC Hughes, England) and one uniquely numbered aluminium or Incoloy band for individual identification. We monitored 21 breeding pairs of colour-banded individuals (in 19 pairs both partners were banded, two pairs contained an un-banded female we were unable to catch). Fifteen of these pairs produced broods which survived > 6 days post-hatch and therefore contributed to our analyses, and four of these pairs contributed two broods each (19 broods in total). Modal brood size was three nestlings (range: 1–4 nestlings). The remaining 14 colour-banded birds were not recorded breeding during the study.

We visited territories on alternate days to ascertain breeding status of the pair, and once nests were initiated, to establish lay and hatch dates of eggs and survival of nestlings. Nestlings hatched asynchronously over one – three days. We made three full-day observations at each nest when the first-hatched nestling was six (n = 17 nests), ten (n = 13 nests), and 14 (n = 12 nests) days old, taking day of hatch as day one (of the 13 nests that survived to fledge, 10 were sampled at all three ages). We banded all nestlings with an aluminium or Incoloy numbered ring either on the evening of day 14 or the morning of day 15.

### Nestling mass gain

We weighed nestlings on a top-pan balance (DS50, Pesola, Baar, Switzerland) twice on each observation day at approximately 6h00 and 18h00. Weighing sessions took ∼ 1 min/nestling after which we immediately returned nestlings to the nest. We calculated diurnal change in body mass (Δ_m_) as a percentage of morning body mass and standardised it for minor variations in timing of morning and evening weighing sessions using the following formula (from [18]):
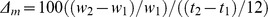



where t_2_–t_1_ is the number of decimal hours between morning (t_1_: ∼6h00) and evening (t_2_: ∼18h00) weighing sessions; w_1_ is mass in morning, and w_2_ is mass in evening.

We marked nestlings on the tarsus or toes with nail varnish for individual identification during the morning weighing session. These markers lasted >12 hours but disappeared between measurement days. This meant we could collect mass change data for individuals at the scale of one day, but could not identify these individuals again on the next observation day. We therefore analysed data as averages per brood to avoid pseudo-replication.

### Provisioning rates

We placed video cameras (Sony HDR-XR160E; Panasonic SDR-S50) on a tripod 2 – 5 m from the nest tree immediately after the morning weighing session on observation days (∼6h00) and retrieved them immediately before the evening weighing session (∼18h00). We extracted data on provisioning rates to nestlings from videos. We divided total number of provisions by number of video-recording hours, then multiplied by 12 to standardise provisioning rate to a 12 hr day. We discarded observations where video recording length was < 10 hrs, which occasionally occurred due to equipment failure or rainstorms, to avoid introducing time-of-day biases into data,.

### Fledging mass, tarsus length and age-at-fledge

We used body mass and tarsus length (measured with Vernier callipers) measurements taken when banding the nestlings as a proxy for fledging body mass and tarsus length [34], [35]. We monitored nests daily after nestlings were banded to establish fledging date.

### Statistical analysis

All analyses were conducted in the R statistical environment [36] using packages lme4 version 0.999375-35 [37], lsmeans version 1.06-05 [38], and MuMIn version 0.13.14, [39].

Factors influencing total provisions per brood per 12-hr day, Δm, and age, tarsus length and body mass at fledging, were investigated by fitting Generalized Linear Mixed Models (GLMMs). Residuals of global and final models were visually inspected to ensure model assumptions were met. Fits of all possible nested models for each analysis were compared using AICc (Akaike’s Information Criteria, adjusted for small samples); models were considered better if they reduced AICc by > 2.

#### Total provisions per day

We analysed provisioning data using a GLMM with Poisson error structure and a log-link function. Four pairs contributed two broods each, but models including the random term brood identity nested within pair identity failed to converge. We therefore removed all observations of one brood each (the brood for which we had fewer observations or, if the number of observations were equal, selected at random) for pairs which had contributed two broods to the dataset and refitted the model including only the random term brood identity.

We included the following fixed factors in the global model t_max_, brood size, nestling age, and all two-way interactions. We removed a single observation with unusually high leverage from the analysis, but this did not influence parameters included in the final model.

Model predictions and 95% CIs were back-transformed by taking the exponential of the sum of the model prediction and the variance component for the random term.

#### Nestling Δm

We analysed nestling Δm data using a GLMM with Gaussian error structure and an identity-link function. Brood size and total provisions per day were strongly correlated. To avoid issues of collinearity, we fitted total provisions per day adjusted for brood size (provisions per nestling  =  provisions per day/brood size)_._

The global model included the random term brood identity nested within pair identity, and fixed factors t_max_, provisions per nestling, nestling age and all two-way interactions. Two observations were removed due to unusually high leverage; this did not influence parameters included in the final model.

#### GLMMs for fledging parameters

We used three approaches to assess the relationship between hot weather during the nestling period and fledging size and age. Firstly, we explored the relationship between mean t_max_ during the nestling period and (a) fledging body mass, (b) fledging tarsus length, or (c) age-at-fledge by fitting separate GLMMs for each response. Each model contained the sole fixed factor mean t_max_ during the nestling period. Models for body mass and tarsus length contained the random term brood identity nested within pair identity. Age-at-fledge models contained one data point per brood (as all nestlings within each brood fledged on the same day) and were fitted with the random term pair identity.

Secondly, we explored whether critical threshold t_max_s (‘t_crit_s’) existed. To do this, we investigated whether increasing numbers of days during the nestling period on which t_max_ > t_crit_ would affect fledgling size and age. For each fledging parameter we fitted a separate model for each 1°C increment in t_max_ from 27°C to 38°C (candidate t_crit_s).We used an identical model structure to that described above, but replaced the fixed effect *mean t_max_* with the *number of days t_max_ > t_crit_*. Effect sizes and 95% CIs for each model were then plotted against candidate t_crit_s to show trends in strength and direction of relationships. This allowed identification of threshold t_crit_s above which fledging parameters were compromised.

Finally, we explored whether fledglings were more vulnerable to hot weather at specific stages of the nestling period, by modelling the same fledging parameters as a function of random terms described above and t_max_ on each day of the nestling period. Effect sizes and 95% CIs were plotted against each day during the nestling period to highlight stages at which high t_max_ most influenced fledging mass, tarsus length, and age. All GLMMs for fledging parameters had a Gaussian error structure and identity link function.

#### Survival analysis

We used the Mayfield estimator [40] to estimate daily survival probability of nests once eggs had hatched:

Daily survival probability  =  1 - (number of failed nests/total number of days survived by all nests)

We estimated daily failure risk as:

Daily failure risk  =  1 - daily survival probability

All data are presented as means (95% CIs), unless otherwise stated. We opted not to report statistical significance (p-values) in order to focus attention on biological relevance of effect sizes, following Garamszegi et al. [41], Nakagawa and Cuthill [42], and others.

## Results

### Daily provisioning rate

We had only one best-fit daily provisioning rate model, which had a model weight of 0.819 (Table 1). The sample size for this analysis was 28 observations of 12 broods from 12 pairs.

**Table 1:** Top five models for total daily provisioning rate.

Model	k	Dev	AICc	ΔAICc	Model weight
Nestling age + brood size + t_max_ + brood size*t_max_	7	68.97	88.57	0.000	0.819
Nestling age + t_max_	5	81.14	93.86	5.293	0.058
Nestling age + brood size + t_max_ + nestling age* t_max_ + brood size* t_max_	9	66.59	94.59	6.019	0.040
Nestling age + brood size + t_max_	6	78.99	94.99	6.419	0.033
Nestling age + t_max_ + nestling age* t_max_	7	76.79	96.39	7.819	0.016

* Dev  =  model deviance. Global model: nestling age + brood size + t_max_ + nestling age* t_max_ + brood size* t_max_ + nestling age*brood size. Random term: brood identity. N = 28 observations of 12 broods from 12 pairs.

The best-fit model contained nestling age, brood size, t_max_, and brood size*t_max_, as well as the random factor brood identity (Table 2).Daily provisioning rates to broods of ten-day old nestlings (mean: 84.9; range: 72.2–100.1) were higher on average than to broods of six- (68.2; 57.4–81.0) or 14-day old (65.7; 55.0–78.4) nestlings.

**Table 2:** Factors affecting total daily provisioning rate, estimates of effect sizes, standard errors (SE), and 95% confidence intervals (95% CI).

Variable	Estimate	SE	95% CI
t_max_	0.05	0.03	–0.01 – 0.11
Brood size	1.30	0.36	0.59 – 2.01
Nestling age:			
six days	2.14	1.01	0.16 – 4.12
ten days	2.36	0.99	0.42 – 4.30
fourteen days	2.10	0.99	0.16– 4.04
Brood size * t_max_	–0.03	0.01	–0.05 – –0.01

* N = 28 observations of 12 broods from 12 pairs. The model was run with a Poisson error structure and log-link function. Effect size estimates are not back-transformed, therefore no units are presented.

There was a negative brood size*t_max_ interaction, such that larger broods experienced a greater reduction in total provisions per day than smaller broods as t_max_ increased. Visual examination of data suggested this was because larger broods received greater numbers of provisions than smaller broods at low t_max_, but at high t_max_ brood size no longer influenced provisioning rate.

### Nestling mass change

**Figure 1:**
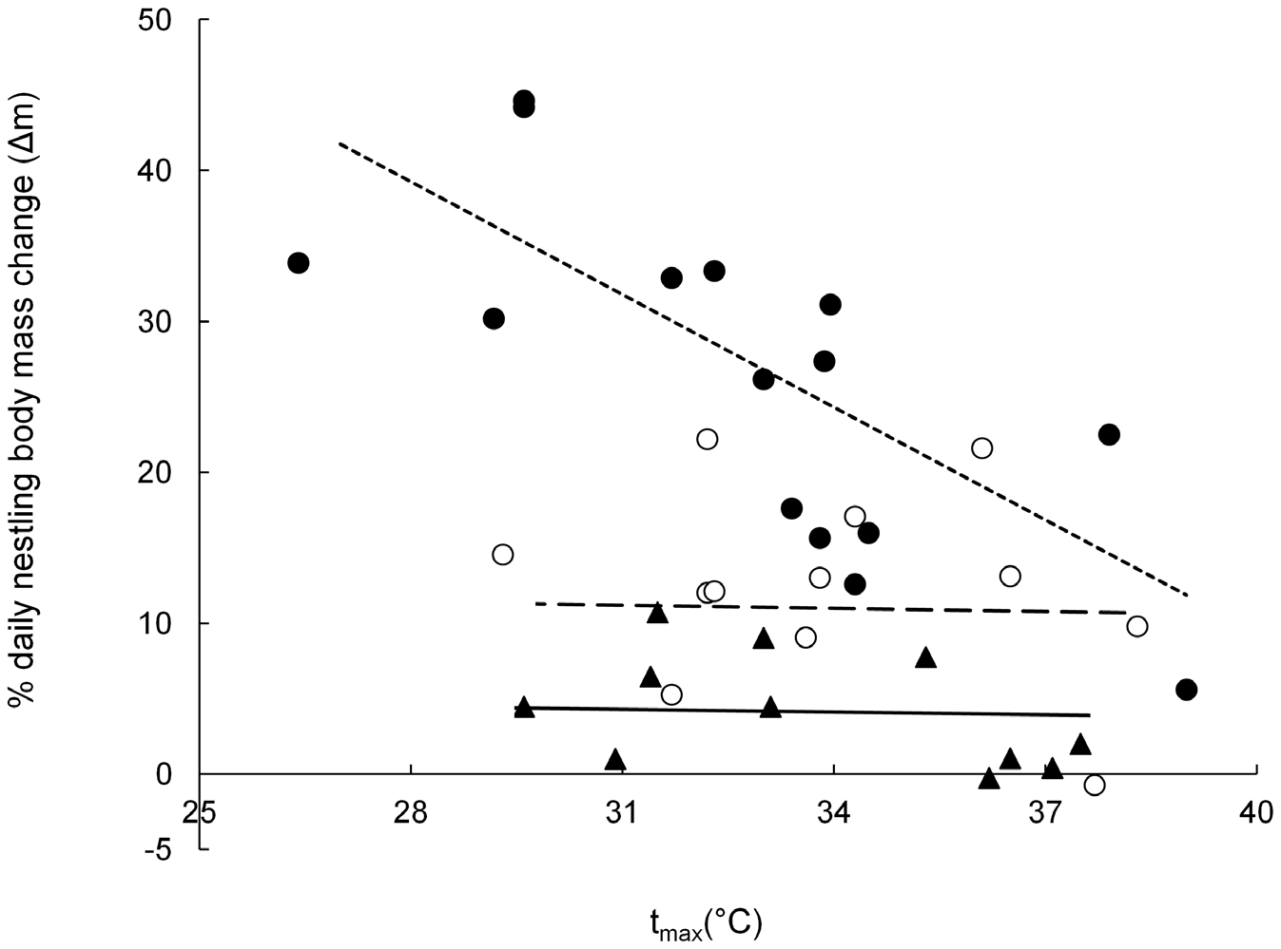
The effect of maximum daily temperature on % daily mass change of nestlings. The negative effect of t_max_ on % daily mass change of nestlings (Δm) was modified by nestling age. Closed circles and dotted line represent six-day old nestlings; open circles and dashed line represent ten-day old nestlings, stars and solid line represent 14-day old nestlings. Each data point represents Δm averaged over all nestlings in a single brood. Lines of best fit were calculated using model predictions from a GLMM at average levels of provisioning and take into account variance caused by random terms.

We had only one best-fit model for nestling Δm, which had a model weight of 0.953 (Table 3). The sample size for this analysis was 38 observations of 18 broods from 14 pairs. This model contained nestling age, provisions per nestling, t_max_, and the interaction between t_max_ and nestling age, as well as the random effect of brood identity nested within pair identity (Table 4). There was a strong negative effect of increasing t_max_ on nestling Δm for six-day old nestlings (where 1°C increase in t_max_ resulted in 2.5% less body mass gain), but increasing t_max_ had a negligible effect on older nestlings (1°C increase in t_max_ resulted in < 0.1% reduction in body mass gain, Fig. 1, Table 4). Increasing numbers of provisions per nestling positively influenced Δm, with each additional provision increasing body mass gain by 0.36% (Table 4). Daily body mass gain of six-day old nestlings (27.5%; 22.1 – 32.9%) was higher on average than ten (13.2%; 9.5 – 16.8%) or 14-day old nestlings (4.3%; 2.0 – 6.5%). In absolute terms, these % body mass gains were equivalent to 2.7 g (2.2 – 3.2 g) for six-day old chicks, 2.7 g (2.0 – 3.3 g) for ten-day old chicks, and 1.2 g (0.5 – 1.9 g) for 14-day old chicks (average morning body mass was 10.8 g (9.6 – 12 g), 22.7 g (20.7 – 24.7 g) and 29.3 g (27.3 – 31.2 g), respectively).

**Table 3:** Top five models for nestling % daily body mass gain (Δm).

Model	k	Dev	AICc	ΔAICc	Model weight
Nestling age + ppn + t_max_ + nestling age*t_max_	10	216.0	244.1	0.000	0.953
Nestling age + ppn + t_max_ + nestling age*ppn + nestling age*t_max_	12	214.9	251.4	7.305	0.025
Nestling age + ppn + t_max_ +nestling age*t_max_ + ppn*t_max_	11	220.8	253.0	8.840	0.011
Nestling age + ppn + t_max_	8	233.4	254.4	10.270	0.006
Nestling age + t_max_ +nestling age*t_max_	9	230.9	255.4	11.260	0.003

* ppn  =  provisions per nestling. Dev  =  model deviance. Global model: nestling age + ppn + t_max_ + nestling age* t_max_ + ppn* t_max_ + nestling age*ppn. Random term: brood identity nested within pair identity. N = 38 observations of 18 broods from 14 pairs.

**Table 4:** Factors affecting nestling % daily mass change (Δm), estimates of effect sizes, standard errors (SE), and 95% confidence intervals (95% CI).

Variable	Estimate	SE	95% CI
t_max_	–0.06	0.56	–1.16 – 1.04
Nestling age:			
six days	97.69	13.42	71.39 – 123.99
ten days	2.10	19.57	–36.26 – 40.46
fourteen days	–5.13	19.78	–43.90 – 33.64
Provisions per nestling	0.36	0.07	0.22 – 0.50
t_max_ *nestling age:			
t_max_ *six days	–2.49	0.40	–3.27 – –1.71
t_max_ *ten days	–0.07	0.56	–1.17 – 1.03
t_max_ *fourteen days	–0.06	0.56	–1.16 – 1.04

* N = 38 observations of 18 broods from 14 pairs. Units for estimates of effect size are % daily body mass change (Δm).

### Relationships between hot weather during the nestling period and fledging parameters

#### Mean t_max_

Mean t_max_ during the nestling period had negative effects on fledging body mass (–1.33 g per 1°C increase in mean t_max_, 95% CI: –2.9 – 0.25) and tarsus length (–0.10 mm per 1°C increase in mean t_max_, 95% CI: –0.69 – 0.49), but 95% CIs were large and included zero in both cases. Modal age-at-fledge was 18 days (range: day 15 to day 21). Mean t_max_ had a positive effect on age-at-fledge (+ 0.70 days per 1°C increase in mean t_max_), but again the 95% CI (–0.31 – 1.71) was large and included zero. Direction of the effect of mean t_max_ on fledging parameters was therefore uncertain, and wide CIs suggest this measure does not capture well the mechanisms underlying variation in fledging parameters.

#### Effects of exceeding t_max_ thresholds

**Figure 2:**
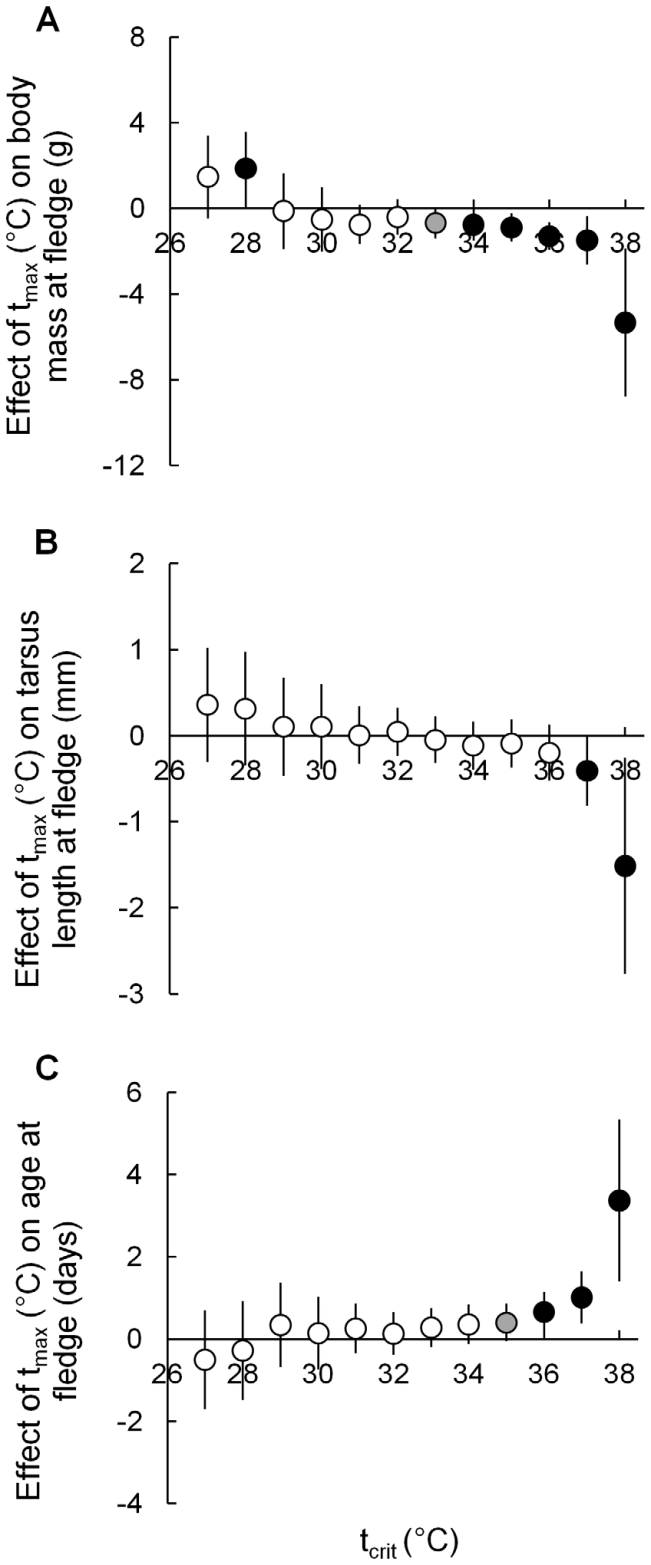
Relationships between numbers of days t_max_ > t_crit_ during the nestling period and fledging parameters. Model estimates and 95% CIs for relationships between number of days on which t_max_ > t_crit_ (plotted on the x-axis) and A: fledging mass; B: fledging tarsus length; and C: age-at-fledge. The y-axis shows the effect on each fledging parameter of a single day during the nestling period on which t_max_ > t_crit_. For example in A: a single day of t_max_ > 38°C will reduce fledgling body mass by 5.3 g. As number and intensity of hot days within the nestling period increases, the size of fledglings decreases (A & B) and nestlings take longer to fledge (C). White circles indicate 90% CIs include zero; grey circles indicate that 90% CIs exclude zero; black circles indicate that 95% CIs exclude zero.

Increasing frequency with which t_max_ > t_crit_ (see Methods) during the nestling period resulted in reduced fledging weight and tarsus length as well as delaying fledging. Threshold t_crit_s exist for all parameters, above which the strength of relationships increased (Fig. 2). For example, the number of days during the nestling period on which t_max_ exceeded t_crit_s ≥ 33°C had negative effects on fledging body mass (90 or 95% CIs for these estimates do not contain zero) which increased in strength as t_crit_ increased (Fig. 2A).

#### Impact of t_max_ at different nestling stages

**Figure 3:**
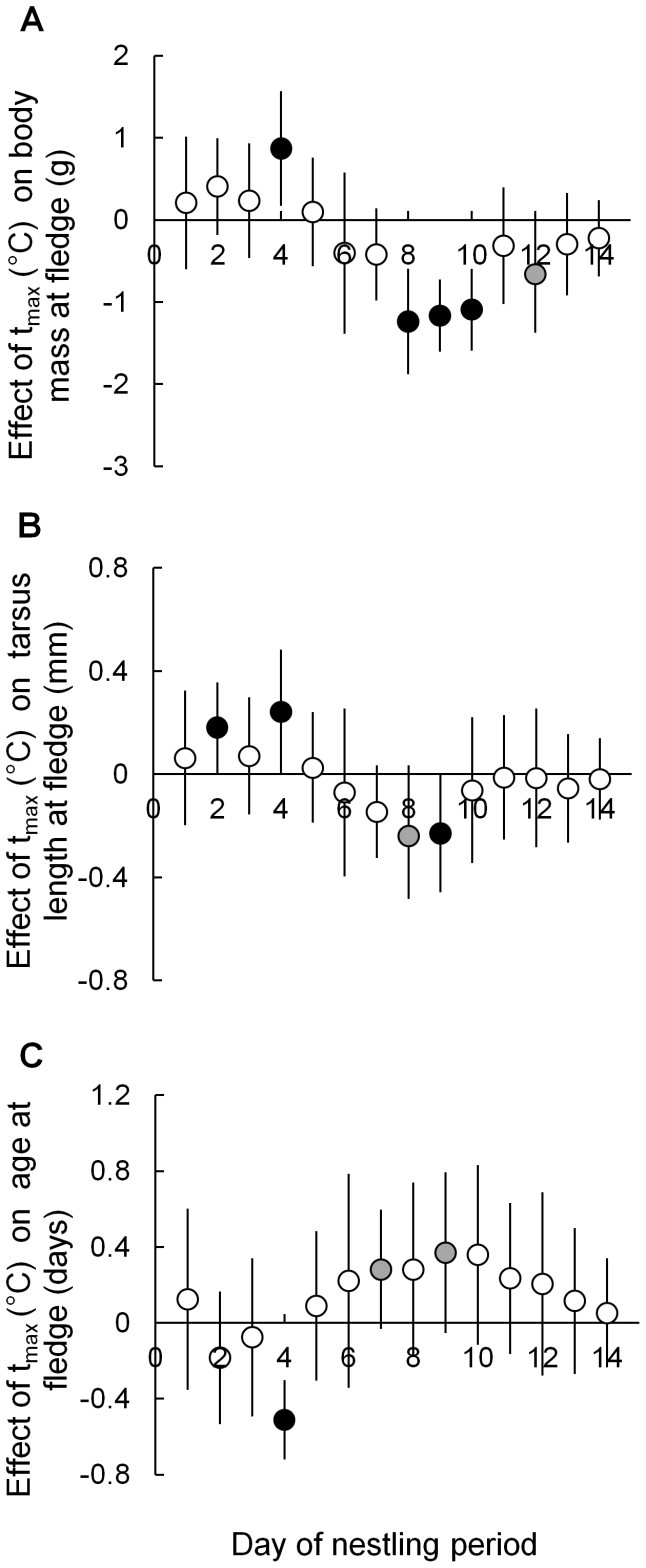
Relationships between t_max_ on each day of the nestling period and fledging parameters. Model estimates and 95% CIs for relationships between t_max_ on each day of the nestling period (day 1  =  day of hatch) and A: fledging mass (g); B: fledging tarsus length (mm); and C: age-at-fledge. The y-axis shows the effect of a 1°C increase in t_max_ on the day of the nestling period indicated on the x-axis. For example in A: a 1°C increase in t_max_ on day eight of the nestling period reduced fledgling body mass by 1.2 g. White circles indicate 90% CIs include zero; grey circles indicate that 90% CIs exclude zero; black circles indicate that 95% CIs exclude zero.

Days with high t_max_ early in the nestling period tended to promote higher body mass and longer tarsi in fledglings, and earlier fledging (Fig. 3). These effects were reversed during the middle of the nestling period, when high t_max_ tended to delay fledging and reduce fledging body mass and tarsus length. High t_max_ had a particularly strong negative effect on fledging body mass on days eight, nine, and ten (Fig. 3A), and on fledging tarsus length on days eight and nine (Fig. 3B). Late in the nestling period (days eleven and following) all t_max_ effects diminished in size (Fig. 3). For all fledgling parameter models, there were 32 fledglings from 13 nests.

### The risks of delaying fledging

Forty-five nests were initiated by colour-banded birds during this study. Of these, twenty-five survived to hatching, and thirteen survived to fledge. The daily post-hatching survival probability of nests was 95.8%. Therefore, risk of nest failure increased by 4.2% per additional day spent in the nest, post-hatching.

## Discussion

Increasing numbers of hot days during the nestling period affected quality, and potentially quantity, of common fiscal fledglings produced, suggesting a mechanism by which predicted temperature increases in the Kalahari [21], [43] could negatively affect populations. We found a strong negative effect of increasing t_max_ on the daily body mass gain of young (six-day old) nestlings. This effect was mediated by reduced parental provisioning rates to larger broods on hotter days, and additionally by direct effects of high temperatures on nestlings. We found only weak evidence for a relationship between mean t_max_ over the entire nestling period, and fledgling body mass, tarsus length and age-at-fledge. Instead, these factors were strongly influenced by frequency with which t_max_ exceeded certain thresholds (t_crit_s). Increasing numbers of days of t_max_ > 33°C during the nestling period resulted in reduced fledgling body mass, increasing number of days of t_max_ > 37°C negatively influenced fledgling tarsus length, and increasing number of days of t_max_ > 35°C positively influenced age-at-fledge. Furthermore, the impact of hot weather on fledging parameters was greatest when high t_max_ days occurred during the middle of the nestling period (∼days 7–10). Thus, ‘hot’ nestling periods, measured in terms of number and timing of hot days (as opposed to the average t_max_), resulted in smaller fledglings which left the nest later. This had implications both for probability of fledging (daily time-dependant mortality risk of nestlings was 4.2%) and potentially for fledgling lifetime fitness [44]–[46].

### Mechanisms underpinning temperature effects on daily nestling body mass gain

Effects of environmental variables (weather conditions, habitat quality) on nestling growth are often mediated by parental provisioning rates [47]–[49]. We found a negative relationship between t_max_ and provisioning rate in common fiscals that interacted with brood size, such that large broods experienced the greatest reduction in provisioning as temperatures increased. Much of the prey provisioned by common fiscals in our study was arthropod invertebrates. Like other ectotherms, invertebrates make behavioural adjustments to regulate body temperature within an optimum range [50]. In cool climates, avian provisioning rates may therefore increase with increasing temperature, as invertebrates become more active [51], [52]. However, above an upper temperature threshold, invertebrates seek out cooler microclimates, retreating underground or into shade. Therefore, reductions in common fiscal provisioning rates on hot days may have been due to reduced prey availability. Alternatively, fiscals may have traded-off provisioning behaviour against their own thermoregulatory requirements, as documented in other desert-dwelling passerines [22], [23]. Further work is needed to assess which of these processes is most important in determining provisioning rates of insectivorous birds during periods of high temperature.

In our study, daily nestling mass gain declined on hot days to a greater extent than could be explained by reductions in provisioning rate alone, suggesting that direct physiological costs of high t_max_ (extra expenditure of energy and water or reduction in the efficiency of physiological processes [15]), may also play a role. We were unable to quantify precisely the relative importance of reduced provisioning vs. direct temperature effects on daily nestling mass gain, for two main reasons. Firstly, we were able to quantify the number of provisions brought to the nest but not biomass delivered. This was due to low quality video from standard definition cameras and lack of data on biomasses of identifiable prey items. Breeding common fiscals are central place foragers (with the nest as the ‘central place’). Central Place Foraging Theory predicts that when costs of foraging are high, parents should return with larger loads [53]. Despite this, conflicting evidence exists to suggest that some avian parents may actually reduce biomass of food loads brought to the nest when foraging is costly [47], [54], [55]. It is therefore uncertain whether our assessment of only provisioning rate and not biomass was more likely to have underestimated or overestimated reductions in food delivery at high temperatures.

Secondly, once provisioning rate was accounted for, the effect of temperature on nestling mass gain was modified by nestling age. Six-day old common fiscals showed stronger reductions in body mass gain on hot days than ten- or 14-day old nestlings. Similar patterns have been shown in other species [28], [29], suggesting that younger nestlings may generally be more vulnerable to temperature effects. Furthermore, six-day old nestlings in our study gained a much higher percentage of their body mass per day on average, than ten- or 14-day old birds, perhaps making temperature-related effects on mass gain easier to detect at this age (measurement errors would be smaller relative to the effect size). Interestingly, there was no interaction between age and provisioning rate on nestling mass gain. This suggests the relative importance of *direct* temperature costs (as opposed to those realised through provisioning) were higher for younger than older nestlings. Studies in controlled environments where provisioning rates and ambient temperature can be varied independently (see [25], [56]) might help to better disentangle these effects.

### Impact of temperature on size and age at fledging

Contrary to expectations, strong effects of high t_max_ on body mass gain of six-day old nestlings were not reflected in their fledging body mass. Instead, high temperatures later in the nestling period (particularly days eight and nine; Fig. 3), had a greater effect on body mass, tarsus length, and age-at-fledge. Growth of avian nestlings usually approximates a sigmoidal curve, with the most rapid growth rates occurring mid-nestling period [46]. Our data suggest the period of most rapid growth had commenced by day six, growth rates were declining by day ten, and nestlings were approaching asymptotic body mass by day 14. Organisms may compensate for suppressed growth during early development by accelerating growth rates when conditions improve, although this can carry costs later in life (reviewed by [57]). Younger nestlings may therefore have been able to compensate for negative effects of high temperatures, both in terms of growth and recovery from dehydration, whereas older nestlings may have had insufficient time to “catch up” prior to fledging.

Lack of strong evidence for an effect of mean t_max_ on fledging size is unsurprising given the differential impacts of high t_max_ on different days during the nestling period. Instead, fledging parameters were affected by the frequency during the nestling period with which t_max_ exceeded critical thresholds. This importance of thresholds, as opposed to mean temperatures, is likely due to the non-linear shape of physiological responses to temperature [15]. For example, for adult common fiscals the upper critical limit of the TNZ is between 35°C and 38°C ambient temperature. Above this critical limit, metabolic expenditure related to thermoregulation increases dramatically. Common fiscals use facultative hyperthermia (raise the body temperature ∼2°C above normal) to reduce water costs of thermoregulation at ambient temperatures > 30°C in the lab [58].To the best of our knowledge, the physiological response of common fiscal nestlings to elevated temperature has never been studied; therefore the upper critical limit of the TNZ for a homeothermic fiscal nestling is unknown. Despite this, we can assume such a threshold exists and may be implicated in the non-linear relationship between nestling-period t_max_s and fledging outcomes we observed

The relationship between ambient temperature in the lab, air temperature in the field and range of environmental temperatures experienced by birds is complex due to the influence of wind, solar radiation, and humidity, and variation in physical characteristics of the birds themselves [59]. However, in absence of wind, environmental temperature in the shade may approximate air temperature [60]. Daily t_max_ probably therefore represents one of the cooler thermal environments available to adult common fiscals at the hottest time of day (on windy days, increased convective heat loss may mean standard operative environmental temperatures (*sensu* [31]) in the shade could be even cooler than air temperature). In this study, environmental temperatures in nests were likely higher than air temperature as many were at least partially exposed to the sun. Despite these complexities, the range of environmental temperatures available to fiscals at the hottest time of day is likely to correlate, to some degree, with t_max._ (i.e. environmental temperatures are likely to be higher overall on high t_max_ days). On hot days with t_max_ near the upper critical limits measured in the lab, fiscals would be likely to encounter a range of environmental temperatures including some above their TNZ. Thermoregulatory costs at such environmental temperatures perhaps played a role in observed reductions in provisioning rates by adult birds. Under such conditions, homeothermic nestlings may also have been obliged to channel resources towards thermoregulation that might otherwise have been used for growth.

### Implications for body size patterns

Thermoregulatory considerations underlie the predictions of Bergmann’s Rule that endotherms should become larger with increasing latitude because a lower surface area to volume ratio helps conserve body heat [61]. In hot environments, smaller body size may therefore confer thermoregulatory advantages through increasing efficiency of convective cooling - provided environmental temperatures remain below body temperature. Interestingly, a recently study of museum specimens documented reductions in body size in Australian passerines in accordance with increasing temperatures over the last century [62], reflecting similar trends observed in Israel [63]. Gardner *et al.* [62] found no ptilochronological evidence of nutritional stress as a driver of the body size reductions, and therefore suggested they might be an adaptive response to climate change, in keeping with Bergmann’s Rule [62], [64]. The effect of temperature we found on fledging size is unlikely to be detectable in feather growth bars of adults after their first post-fledging moult. We therefore suggest a third potential explanation: direct (physiological) and indirect (nutritional) effects of higher temperatures during critical stages of nestling growth, which translate into reductions in adult body size.

### Implications for individual fitness and populations under warming trends

Reduced fledging size in birds is often correlated with reduced survival, recruitment into the breeding population, fecundity, and reproductive success [45], [65]–[68]; however see [69], [70]. Temperature-driven variation in body size is therefore likely to have far-reaching implications for life-histories of common fiscals that experience ‘hot’ weather conditions while in the nest. The southern Kalahari and surrounding areas in north-western South Africa are undergoing among the fastest rates of warming in the region [32], with the implication that such ‘hot’ nestling periods will become more frequent. This is especially the case because arid-zone birds tend to breed in response to the onset of rainfall [71] and are therefore unlikely to be able to advance breeding dates to avoid the hottest part of the season. As warming continues, high temperatures during the breeding season may affect the absolute fitness of increasing proportions of individuals within each cohort of fledglings, with consequences for the maintenance of the southern Kalahari fiscal population.

We believe that investigations of the shape of the relationship between t_max_ and correlates of fitness, such as presented here, are of great importance for predicting species’ responses to climate change. In this study, we discovered biologically meaningful critical threshold t_max_s which if exceeded during the nestling period, are likely to affect the fitness of common fiscal fledglings. It is probable that such thresholds exist in other species as well, due to the non-linear nature of physiological responses to temperature. Identification of these thresholds will provide solid data for use in predicting the impacts of past and future climate change on populations and species.
